
JOURNAL INFORMATION
==============================
NLM Title Abbreviation: Intensive Care Med Exp
Journal ID: Intensive Care Med Exp
NLM Journal ID: 101645149

Intensive Care Medicine Experimental

EISSN: 2197-425X
Publisher: Springer

ARTICLE INFORMATION
==============================
PMCID: PMC13653028
PMID: 42855639
DOI: 10.1186/s40635-026-00962-w
Article ID: 962
Article version: 1
Subjects: Meeting Abstracts

ESICM LIVES 2026

Electronic publication date: 2026 Oct 9
Publication date: 2026 Oct
Volume: 14
Issue: Suppl 1
Issue sponsor: Publication of this supplement has been supported by ESICM.
Electronic Location ID: 131
Copyright: © The Author(s) 2026
Copyright year: 2026
License: Open Access This article is licensed under a Creative Commons Attribution 4.0 International License, which permits use, sharing, adaptation, distribution and reproduction in any medium or format, as long as you give appropriate credit to the original author(s) and the source, provide a link to the Creative Commons licence, and indicate if changes were made. The images or other third party material in this article are included in the article's Creative Commons licence, unless indicated otherwise in a credit line to the material. If material is not included in the article's Creative Commons licence and your intended use is not permitted by statutory regulation or exceeds the permitted use, you will need to obtain permission directly from the copyright holder. To view a copy of this licence, visit http://creativecommons.org/licenses/by/4.0/.
License URL: https://creativecommons.org/licenses/by/4.0/

Conference name: ESICM LIVES 2026
Conference location: Lisbon, Portugal
Conference date: 10-14 October 2026
issue-copyright-statement: © European Society of Intensive Care Medicine and Springer Nature Switzerland AG 2026

==============================
Publisher's Note

Springer Nature remains neutral with regard to jurisdictional claims in published maps and institutional affiliations.

